# Generation of Human Antigen-Specific Monoclonal IgM Antibodies Using Vaccinated “Human Immune System” Mice

**DOI:** 10.1371/journal.pone.0013137

**Published:** 2010-10-04

**Authors:** Pablo D. Becker, Nicolas Legrand, Caroline M. M. van Geelen, Miriam Noerder, Nicholas D. Huntington, Annick Lim, Etsuko Yasuda, Sean A. Diehl, Ferenc A. Scheeren, Michael Ott, Kees Weijer, Heiner Wedemeyer, James P. Di Santo, Tim Beaumont, Carlos A. Guzman, Hergen Spits

**Affiliations:** 1 Department of Vaccinology and Applied Microbiology, Helmholtz Centre for Infection Research (HZI), Braunschweig, Germany; 2 Department of Cell Biology and Histology, Academic Medical Center of the University of Amsterdam (AMC-UvA), Center for Immunology Amsterdam (CIA), Amsterdam, The Netherlands; 3 AIMM Therapeutics, Amsterdam, The Netherlands; 4 Cytokines and Lymphoid Development Unit, Institut Pasteur, Paris, France; 5 INSERM U668, Institut Pasteur, Paris, France; 6 Clinic for Gastroenterology, Hepatology, and Endocrinology, Hannover Medical School, Twincore Centre of Experimental and Clinical Infection Research, Hannover, Germany; New York University, United States of America

## Abstract

**Background:**

Passive transfer of antibodies not only provides immediate short-term protection against disease, but also can be exploited as a therapeutic tool. However, the ‘humanization’ of murine monoclonal antibodies (mAbs) is a time-consuming and expensive process that has the inherent drawback of potentially altering antigenic specificity and/or affinity. The immortalization of human B cells represents an alternative for obtaining human mAbs, but relies on the availability of biological samples from vaccinated individuals or convalescent patients. In this work we describe a novel approach to generate fully human mAbs by combining a humanized mouse model with a new B cell immortalization technique.

**Methodology/Principal Findings:**

After transplantation with CD34^+^CD38^−^ human hematopoietic progenitor cells, BALB/c Rag2^−/−^IL-2Rγc^−/−^ mice acquire a human immune system and harbor B cells with a diverse IgM repertoire. “Human Immune System” mice were then immunized with two commercial vaccine antigens, tetanus toxoid and hepatitis B surface antigen. Sorted human CD19^+^CD27^+^ B cells were retrovirally transduced with the human B cell lymphoma *(BCL)-6* and *BCL-XL* genes, and subsequently cultured in the presence of CD40-ligand and IL-21. This procedure allows generating stable B cell receptor-positive B cells that secrete immunoglobulins. We recovered stable B cell clones that produced IgM specific for tetanus toxoid and the hepatitis B surface antigen, respectively.

**Conclusion/Significance:**

This work provides the proof-of-concept for the usefulness of this novel method based on the immunization of humanized mice for the rapid generation of human mAbs against a wide range of antigens.

## Introduction

Hyper-immune sera containing polyclonal immunoglobulins (Igs) have been widely used in both therapeutic and prophylactic clinical settings [1]. However, the use of polyclonal sera was associated with several problems, such as the stimulation of allergic reactions, low reproducibility between clinical batches and high off-label use, which finally caused a decline in their use [2]. The advent of technologies to make monoclonal antibodies (mAbs) derived from animals, especially mice, has overcome many of the problems associated with the use of polyclonal sera. The technology to make monoclonal cell lines of antibody-producing cells by fusing antibody producing plasma cells with myeloma cells was described for the first time in 1975 by Milstein and Kohler [3]. The therapeutic potential of mAbs was immediately recognized and in 1980 the first mAb, OKT3, was approved for therapeutic applications. This antibody inactivates T cells, thereby preventing rejections of organ transplants [4]. However, because of the animal origin of the first generation of mAbs that were used in clinical trials, human subjects treated with these antibodies developed vigorous immune reactions against the animal proteins, which were thereby eliminated preventing their therapeutic actions [5]. To overcome these problems technologies were developed to diminish the immunogenicity of mouse antibodies by replacing part or the complete mouse antibody backbone by its human equivalent, first generating chimeric, and subsequently fully humanized antibodies [6]. In a parallel approach transgenic mice bearing the human Ig region were created to obtain fully human antibodies following immunization. The use of these mice obviates the elaborate molecular engineering of antibodies that is needed to humanize antibodies generated in wild-type mice, however, the maturation process of the mouse B cells expressing human Igs is different from that of fully human B cells [7].

Immortalization of B cells from immune humans seems to be the logical strategy to avoid these problems. However, the methods to achieve this goal have showed low efficiencies, although some progress has recently been reported [8], [9]. Nevertheless, the major disadvantage of human B cells immortalization is the need for cells from either vaccinated individuals or patients who had recovered from an infection. Thus, to fully exploit the Ig repertoire of human B cells in an in vivo setting, we explored the possibility to raise mAbs following *de novo* induction of human B cell responses in mice carrying elements of the human immune system (HIS). HIS mice are generated by engrafting immunodeficient mice with human hematopoietic stem cells (HSC) with or without human lymphoid tissues from fetal origin [10], [11], [12]. In particular, mice deficient for the recombinase activating gene-2 (*Rag2*) and the common gamma chain of the IL-2 receptor (*Il2rg*) on a BALB/c or a non-obese diabetic (NOD) background are permissive for human HSC xenografts. Inoculation of newborn mice from these strains with human HSC of fetal or umbilical cord blood origin gives rise to robust engraftment of a number of immune cells, including T, B, NK and dendritic cells. In this work, we describe a convenient approach to generate fully human mAbs based on the immunization of BALB/c Rag2^−/−^IL-2Rγc^−/−^ engrafted with human CD34^+^CD38^−^ HSC [13], [14]. To this end, HIS mice were immunized with commercial vaccines against hepatitis B virus (HBV) and tetanus. Following immunization, human CD19^+^ B cells were sorted based on surface CD27 expression, as a marker of memory phenotype, and the isotype of surface Igs. The sorted B cell populations were immortalized *in vitro* by retroviral transduction with human B cell lymphoma *(BCL)-6* and *BCL-XL* genes and antigen-specific B cell clones were established and characterized.

The obtained results provided the proof-of-concept for the usefulness of this generic approach based on HIS mice combined with immortalization of human B cells for the rapid and inexpensive development of human mAbs against a wide range of antigens.

## Materials and Methods

### Ethics statement

The use of fetal liver tissue obtained from elective abortions with gestational age ranging from 14 to 20 weeks was approved by the Medical Ethical Committee of the AMC-UvA and was contingent on informed written consent.

### Generation of HIS-mice

BALB/c Rag2^−/−^IL-2Rγc^−/−^ mice were bred and maintained in individual ventilated cages, and fed with autoclaved food and water. HIS mice were generated as previously described [13], [14], [15], [16], with the approval of the Animal Ethical Committee of the AMC-UvA (permit number DHL-100970). In brief, human fetal livers were obtained from elective abortions with gestational age ranging from 14 to 20 weeks. Magnetic enrichment of CD34^+^ cells (>98% pure) was performed by using the CD34 Progenitor Cell Isolation kit (Miltenyi Biotech), after preparation of single cell suspensions and isolation of mononuclear cells by density gradient centrifugation over Lymphoprep (Axis Shield). Finally, newborn (<5 days old) sub-lethally irradiated (3.5 Gy) BALB/c Rag2^−/−^IL-2Rγc^−/−^ mice were injected via intra-hepatic route with 5–10×10^4^ sorted CD34^+^CD38^−^ human fetal liver hematopoietic stem cells in 30 µl. All manipulations of HIS mice were performed under laminar flow. Cell suspensions were prepared in RPMI medium supplemented with 2% fetal calf serum (FCS).

### BCR V_H_ and CDR3 immunoscope analysis

Twelve to sixteen weeks after CD34^+^CD38^−^ HSC engraftment, HIS mice were killed and single cell suspensions of splenocytes were prepared. Red cells lysis was performed in 1 ml of red cell lysis buffer (Sigma) for 10 min. Splenocytes were washed, resuspended in 600 µl of RLT lysis buffer (Qiagen) and homogenized by passing through a 21-gauge needle several times using RNase free syringes. RNA was prepared using RNeasy mini kits (Qiagen) according to manufactures instructions. BCR V_H_ immunoscope was performed as previously described [17]. Briefly, cDNA was prepared and real-time PCR performed by combining primers for the different V_H_ chains (V_H_1-7) and specific fluorochrome-labeled probes against the different constant regions (C_H_μ, C_H_α and C_H_γ). An additional four PCR cycles ‘run-off reactions’ were then performed on the PCR products using fluorescent primers specific for the constant regions (Fcμ, Fcα and Fcγ). Products were gel separated to determine CDR3 lengths. Analysis of six individual HIS-mice containing greater than 30% human chimerism in the spleen was performed. The number of human CD19^+^ B cells in chimeric spleens ranged from 5–12×10^6^.

### Immunization protocol and sample collection

Eight weeks after HSC transplantation, blood was taken from HIS mice to verify the level of engraftment by flow cytometry, as described elsewhere [18]. HIS mice with a good level of human reconstitution (>20% hCD45^+^ cells) were immunized by intramuscular route (biceps femoris) using a 29G needle, three times on weeks 14, 16 and 18 with either 100 µl of the HBV vaccine (Engerix-B, GlaxoSmithKline) or 50 µl of tetanus toxoid (TT) containing vaccine (Tetanus vaccine, The Netherlands Vaccine Institute). These amounts correspond to 1/10 of the normal human dose. Negative controls received the same volume of PBS buffer. Two weeks after the last immunization, HIS mice were exsanguinated under isofluran/oxygen narcosis. Spleens and mLN were removed aseptically and cellular suspensions were prepared. The BM cells were isolated from the femur and tibia.

### Flow cytometry analysis and B cell sorting

Cell suspensions were labeled with FITC, PE, PerCP-Cy5.5, PE-Cy7, APC or APC-Cy7 coupled anti-human mAb targeting the following cell surface markers: CD1a (T6-RD1) and CD38 (CLT16) from Beckman Coulter; CD3 (SK7), CD4 (SK3), CD8 (SK1), CD19 (HIB19), CD38 (HIT2), CD45 (2D1 and HI30), CD45RA (HI100), CD138 (MI15), IgM (G20-127), IgD (IA6-2), IgG (G18-145) and CCR7 (3D12) from BD Biosciences; CD27 (LT27) from AbD-Serotec; CD27 (LG.7F9) from eBioscience. TT-specific B cells were also occasionally stained with PE-coupled TT, kindly provided by Dr. Andreas Radbruch (German Rheumatism Research Center, Berlin, Germany). Dead cells were excluded based on DAPI incorporation. All washings and reagent dilutions were done with PBS containing 2% FCS and 0.02% NaN_3_. Stained cells were analyzed with an LSR-II interfaced to a FACS-Diva software system (BD Biosciences). Cell sorting of B cell subsets were performed on HIS mouse BM and spleens using a FACS-Aria cell sorter interfaced to a FACS-Diva software system (BD Biosciences). For these experiments, all washings and reagent dilutions were done with 2% FCS supplemented PBS without NaN_3_.

### Retroviral transduction, culture and Ig-V_H_ sequence analysis of human B cell clones

The human *BCL6* [19], [20] and *BCL-XL* [21] encoding cDNAs were further cloned in a LZRS retroviral expression vector, around a T2A cleavage-promoting peptide sequence and upstream a cassette containing an internal ribosome entry site (IRES) and the gene encoding GFP. We therefore obtained a LZRS vector in the following configuration: BCL6-T2A-BCLXL-IRES-GFP [9]. Transfection of Phoenix-GALV packaging cells and virus production were performed as previously described [22]. Before retroviral transduction, memory B cells were activated on γ-irradiated (50 Gy) mouse L cell fibroblasts stably expressing CD40L (CD40L-L cells) in the presence of 25–50 ng/ml recombinant mouse interleukin-21 (rmIL-21, R&D systems) for 36 h [19]. The B cells were washed, mixed with retroviral supernatants in Retronectin-coated plates (Takara), centrifuged at room temperature for 60 min at 360 g, and subsequently incubated with the retroviruses at 37°C, 5% CO_2_ for 6–8 h. Transduced B cells were maintained in co-cultures using CD40L-L cells (10^5^ cells/ml) and in standard IMDM (Gibco) culture medium supplemented with 8% fetal bovine serum (FBS; HyClone), penicillin/streptomycin (Roche) and 25 ng/ml rmIL-21.

The analysis of human Ig-V_H_ sequences was performed as follows. Total RNA was isolated from approximately 5×10^5^ monoclonal B cells with Trizol (Invitrogen). The cDNA was generated and subjected to PCR with primers specific to the different V_H_ family members. PCR products were sequenced to determine the CDR3 region of the different clones. Sequence analysis was performed using BigDye Terminator chemistry (Applied Biosystems Inc.) and CodonCode Aligner software.

### ELISA screening for antigen-specific B cells

The plasma harvested from HIS mice (7 days after the first and second immunization; 10 days after the third immunization) and B cell clone culture supernatants were screened by ELISA for the presence of total human IgM, total human IgG and antigen-specific antibodies. Measurement of total IgM and IgG was performed by coating 96-well plates either with AffiniPure F(ab')_2_ fragment goat anti-human IgM (Fc5μ-specific, Jackson ImmunoResearch) or AffiniPure goat anti-human IgG (Fcγ fragment-specific; Jackson ImmunoResearch). Control human serum protein calibrator (Dako) with known IgM (0.8 mg/ml) and IgG (10.4 mg/ml) concentrations was used as a standard to be compared to the samples. For the detection of antigen-specific antibodies, 96-well plates were coated either with tetanus vaccine (Nederlands Vaccin Instituut) or Engerix B (GlaxoSmithKline) (10× diluted in PBS) for 1 h at 37°C or overnight at 4°C. Alternatively, Ridascreen Tetanus IgG ELISA plates (Biopharm) were also used to screen for TT-specific antibodies. After coating, the plates were washed in ELISA wash buffer (PBS, 0.5% Tween-20). A PBS solution containing 4% of milk was used as a blocking agent, before adding serial dilution of HIS mouse plasma (starting at a dilution of 1∶5) or cell culture supernatants (starting at a dilution of 1∶2). Enzyme-conjugated detection antibodies were added at a dilution of 1∶2500 for HRP-conjugated anti-IgG and a dilution of 1∶5000 for HRP-conjugated anti-IgM (both from Jackson ImmunoResearch). Then, TMB substrate/stop solution (Biosource) was used for the development of the ELISA assay.

### Statistical analysis

Statistical analyses were performed using GraphPad Prism version 5.02 for Windows (GraphPad Software). Data were subjected to two-tailed unpaired Student *t* test analysis. The obtained *p* values were considered significant when p<0.05.

## Results

### HIS mice contain a large B cell IgM repertoire

**Figure 1:**
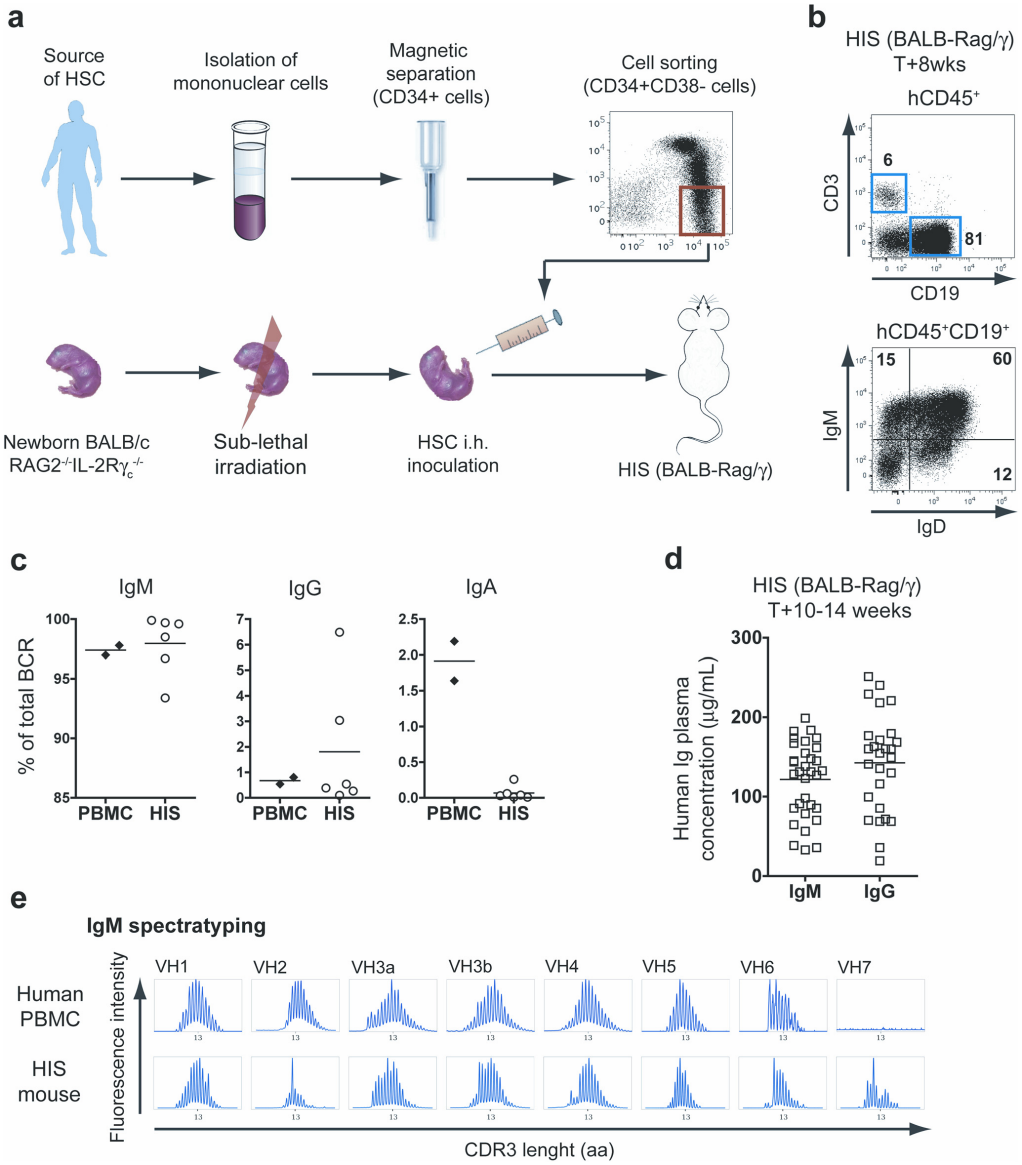
Characterization of B cells in naïve HIS mice. (A) HIS mice are generated by transplanting sorted human CD34^+^CD38^−^ HSC-enriched cell fraction into conditioned (3.5 Gy irradiation) newborn Rag2^−/−^ IL-2Rγc^−/−^ mice. After 6 to 8 weeks post-transplantation, human lymphoid and myeloid cell subsets are found in the blood and lymphoid organs of the reconstituted animals. (B) The top panel shows the flow cytometry analysis of human T (CD3^+^) and B (CD19^+^) cells found in the spleen of 8-week old HIS mice. The expression of IgM and IgD on the surface of B cells is depicted on the low panel. (C) The relative proportion of B cells expressing an IgM, IgG or IgA BCR was determined by quantitative PCR for both control PBMC and HIS splenocytes. Percentages represent the frequency of IgM, IgG and IgA (C_H_μ, C_H_γ and C_H_α) containing V_H_ PCR products out of the total V_H_ PCR products from each spleen. The horizontal bars indicate the mean value. (D) The graph shows the concentration of total IgM and IgG (mean values: horizontal bars) in the plasma of 10- to 14-week old naive HIS mice. (E) The naive IgM B cell repertoire of HIS mice was evaluated on splenocytes by performing a BCR immunoscope for each V_H_ family. The profiles obtained with control human PBMC are also shown.

We have generated HIS mice by transplanting human HSC into alymphoid BALB/c Rag2^−/−^IL-2Rγc^−/−^ newborn mice (**Figure 1A**). As reported previously, multilineage human hematopoietic reconstitution is observed in HIS mice, which demonstrate human thymopoiesis, B cell differentiation, NK cell and plasmacytoid dendritic cell development, and myelopoiesis [10], [13], [14], [15], [16]. Human immune cells accumulate in lymphoid tissues, and several B cell subsets are observed in HIS mice (**Figure 1B**). We analyzed the human B cell repertoire present in naive HIS mice by using B cell receptor (BCR) immunoscope analysis based on quantitative PCR of Ig variable (V_H_) and constant (C_H_) region gene segments [17]. Due to the lack of human spleen samples, the cells isolated from HIS mouse spleens, which contained sufficient numbers of human B cells to perform the immunoscope analysis, were compared to control human peripheral blood mononuclear cells (PBMC) samples, which were considered acceptable for the purpose of the performed comparison. We observed that IgM-expressing B cells as well as Ig isotype-switched B cells are found in naive HIS mice (**Figure 1C**). The vast majority of B cells of HIS mice expressed an IgM (97.9±1.0%), whereas IgG (1.8±1.0%) and IgA (0.07±0.04%) expressing B cells represented minor populations. Only the frequency of IgA-expressing B cells was found significantly higher in control human PBMC samples (p<0.0001). At 10–14 weeks post-transplantation (i.e. in steady state conditions), the human Ig concentrations in the blood were 122±8 µg/ml (IgM) and 143±12 µg/ml (IgG) (**Figure 1D**), as previously reported [8], [16]. In comparison, the normal range for Ig concentration in healthy humans is 400–3100 µg IgM/ml and 7200–14700 µg IgG/ml. In brief, despite a low frequency of IgG-expressing cells, both human IgM and IgG accumulated in the plasma of ∼3 month-old HIS mice to levels representing around 10% and 1% of adult human IgM and IgG concentrations, respectively.

We further examined the antigen receptor repertoire diversity in HIS mice, by determining the length of CDR3 hypervariable regions for each Ig-V_H_ gene family. The analysis of CDR3 length distribution of individual HIS mouse splenocytes showed that IgM repertoires are undistinguishable from normal human PBMC IgM repertoires, as measured by the BCR immunoscope analysis (**Figure 1E**). This observation suggests that HIS mice contain a broad variety of naive IgM^+^ B cell clones. The V_H_-family usage was large and similar to control human PBMC (**Table 1**). The BCR immunoscope analysis was also performed for IgG and IgA repertoires and we observed more restricted repertoires, as expected from B cells undergoing clonal selection and Ig class switch recombination (**Figure S1**).

**Table 1:** Average V_H_ family usage for IgM in HIS (BALB-Rag/γ) mice and control human PBMC.

	HIS (BALB-Rag/γ)	Human PBMC
VH1	0.11±0.03	0.40±0.07
VH2	0.02±0.01	0.05±0.04
VH3a	10.2±3.5	3.5±0.6
VH3b	75.1±4.4	79.9±0.1
VH4	12.5±3.2	11.5±1.7
VH5	0.03±0.01	0.07±0.03
VH6	0.02±0.01	0.030±0.002
VH7	0.0001±0.0002	0.02±0.01

The data from HIS mice were generated with B cells isolated from the spleen of naïve animals. The values are expressed as the relative frequency (%) of each IgM-V_H_ family.

### Immunization of HIS mice with HBV and tetanus vaccines results in the generation of antigen-specific antibody responses

Since HIS mice contained broad naïve B cell repertoires, we analyzed the induction of human antigen-specific B cell responses after immunization with commercially available human vaccines. We designed a vaccination protocol based on repeated intra-muscular immunizations (3 injections with 2-week intervals) of 10–14-week old HIS mice with vaccines containing hepatitis B surface antigen (HBsAg) or TT. Seven days after the last immunization mice were sacrificed, the blood and the lymphoid organs were harvested, and the phenotype and function of human cells was analyzed.

**Figure 2:**
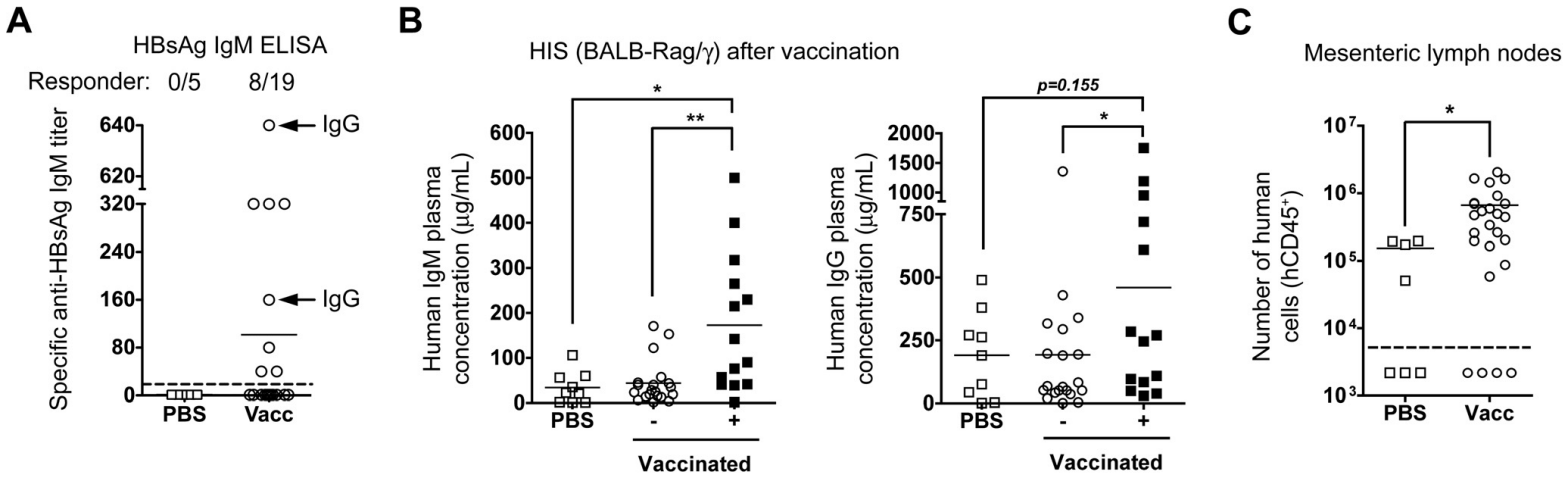
Vaccination of HIS mice. (A) The graph shows the titers of HBsAg-specific IgM measured by ELISA in PBS-injected and HBsAg-vaccinated HIS mice, 10 days after the third i.m. injection. Vaccinated mice are designated as responder animals when antigen-specific antibody titers are above the cut-off value (doted horizontal line). Responder animals with a detectable antigen-specific IgG response are indicated by the horizontal arrows. (B) The graphs show the concentration of total IgM (top graph) and IgG (bottom graph) in the plasma of PBS-injected (open squares) or vaccinated HIS mice (pool of HBsAg- and TT-vaccinated) at the end of the vaccination protocol (mean values: horizontal bars). The vaccinated are separated between non-responder (- columns; open circles) and responder (+ columns; closed squares) mice. (C) The graph shows the number of human cells (hCD45^+^) harvested in mLN of PBS-injected vs. vaccinated (TT and HBsAg) HIS mice. The mLN were not detected in 43% of mock-injected mice, as compared to 16% of vaccinated animals (symbols placed under the doted line). The animals without detectable mLN were excluded to calculate the average number of human cells per group (horizontal bar). * p<0.05; ** p<0.01.

All HIS mice showed human reconstitution (>20% hCD45^+^ cells) in the blood before starting the immunization protocol, which correlated with human engraftment in lymphoid organs. Overall, 42% of HBsAg-vaccinated (8 out of 19 vaccinated animals) and 40% of TT-vaccinated (6 out of 15) HIS mice showed significant production of antigen-specific IgM antibodies, as detected by ELISA (**Figure 2A**). We performed a kinetic monitoring of antigen-specific plasma Ig levels in individual HBsAg-vaccinated responder HIS mice and we observed that after the first immunization antigen-specific Igs were rarely detected. In contrast, after the second immunization antigen-specific IgM was detected, which steadily increased after the third immunization with approximately 25–40% of responder mice also showing an antigen-specific IgG response (**Figure 2A**). This suggests that repeated vaccination leads to enhanced antigen-specific antibody production. The responder mice exhibited higher total IgM (173±41 µg/ml) and total IgG (459±140 µg/ml) concentrations in their plasma, as compared to PBS-injected (IgM: 37±12 µg/ml; IgG: 191±58 µg/ml) and non-responder vaccinated (IgM: 44±11 µg/ml; IgG: 192±67 µg/ml) animals (**Figure 2B**).

At the end of the immunization protocol, vaccinated animals showed significantly higher numbers of hCD45^+^ cells in all organs (i.e. spleen, bone marrow (BM) and mesenteric lymph nodes (mLN)) in comparison to mock-injected control mice. Responder HIS mice exhibited higher numbers of human T and B cells in the spleen, as well as T cells in the BM (**Table 2** **; Table S1**), suggesting that the vaccination protocol had a positive impact on the accumulation of human B and T cells. Moreover, the mLN isolated from vaccinated HIS mice contained 4 to 5-fold more hCD45^+^ cells than those of control animals (**Figure 2C**), suggesting that the mLN structure might play a role in eliciting an immune response in the HIS mice.

**Table 2:** Human cell numbers measured in the spleen of vaccinated HIS mice.

	Human cells (CD45^+^)		
Groups	Total	B cells (CD19^+^)	T cells (CD3^+^)
	Absolute number (×10^6^)	Absolute number (×10^6^)	Absolute number (×10^6^)
**Controls (n = 10)**	0.72±0.27	0.25±0.16	0.43±0.15
**Vaccinated (n = 34)**	3.63±0.75 **	2.03±0.46**	1.45±0.41 *n.s.*
*Responders (n = 14)*	5.63±1.54 **	3.06±0.96**	2.44±0.87 *
*non-responders (n = 20)*	2.06±0.45*	1.28±0.35**	0.59±0.20 *n.s.*

The data from the TT and HBsAg vaccination experiments are pooled. Values are expressed as a mean (± S.E.M.) number of human cells, human B cells and human T cells found in control (PBS-injected) or vaccinated animals. The vaccinated animals were further distributed between responder and non-responder animals, as determined by antigen-specific ELISA. Statistical analysis was performed on the cell numbers harvested from vaccinated animals, as compared to the control group (* p<0.05; ** p<0.01).

### Generation of antigen-specific monoclonal B cell lines from vaccinated HIS mice

**Figure 3:**
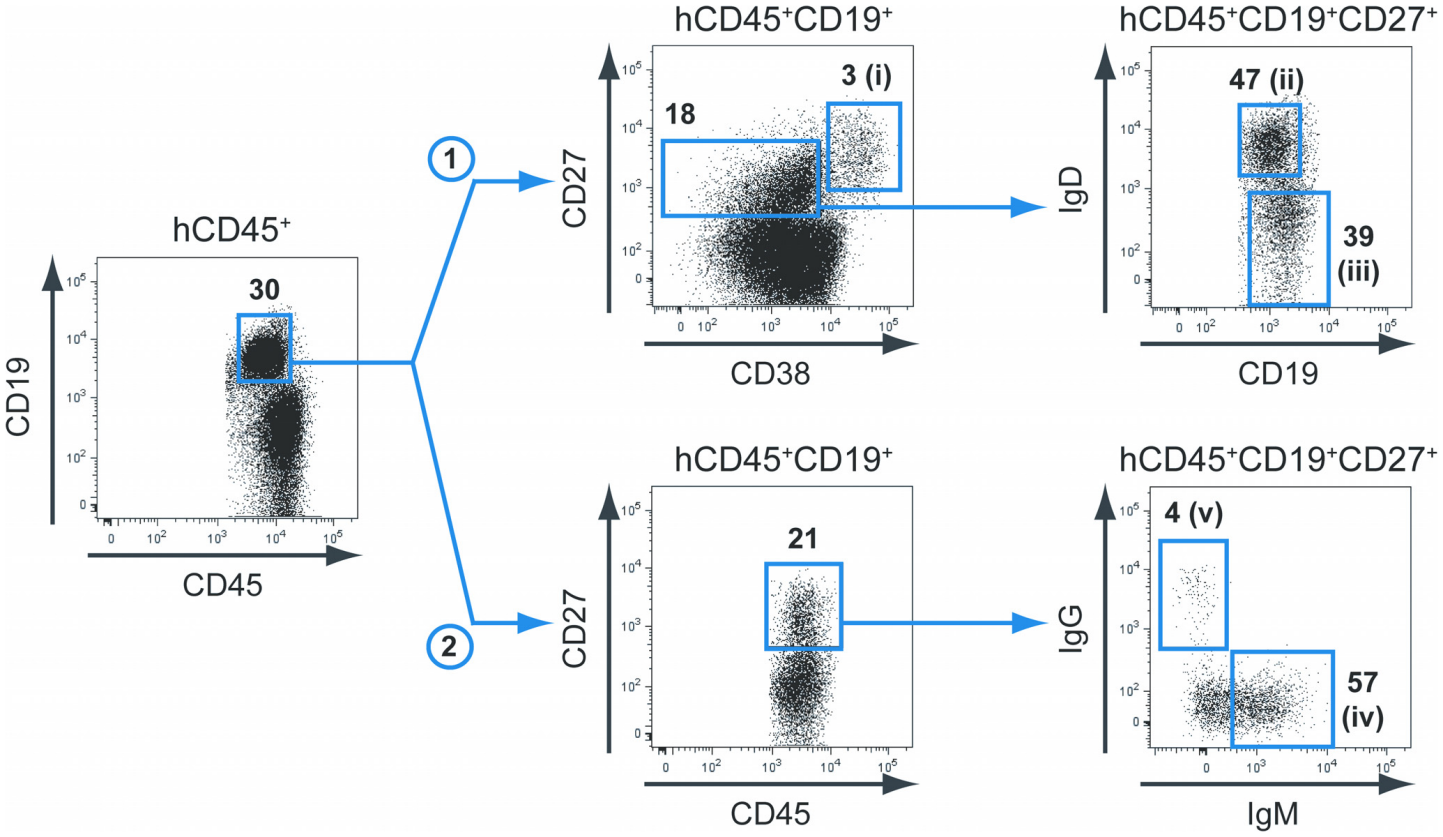
Memory B cell sorting strategies. Subsets of human B cells (CD45^+^CD19^+^) were sorted from BM and spleens of vaccinated HIS mice. The strategy 1 (upper part) was used on 3 responder HBsAg-vaccinated animals and gave rise to the following B cell subsets: (i) CD27^hi^CD38^hi^, (ii) CD27^+^CD38^lo/int^IgD^+^, and (iii) CD27^+^CD38^lo/int^IgD^−^. The strategy 2 (lower part) was used on 3 responder TT-vaccinated animals and gave rise to the following B cell subsets: (iv) CD27^+^IgM^+^IgG^−^, and (v) CD27^+^IgM^−^IgG^+^. The respective frequency and name (from i to v) of each sorted B cell subsets is indicated in the corresponding flow cytometry dot plot.

In humans, the CD27^+^ memory B cell population contains the majority of antigen-experienced B cells [23], [24], and we reasoned that the same should be true in vaccinated HIS mice. We therefore cell sorted several different CD19^+^CD27^+^ B cell subsets from individual HIS mice. We used two strategies to isolate the following human B cell (CD45^+^CD19^+^) subsets from BM and spleens of vaccinated HIS mice: (i) CD27^hi^CD38^hi^, (ii) CD27^+^CD38^lo/int^IgD^+^, and (iii) CD27^+^CD38^lo/int^IgD^−^ on the one hand (**Figure 3** – strategy 1, HBsAg vaccination); and (iv) CD27^+^IgM^+^IgG^−^ and (v) CD27^+^IgM^−^IgG^+^ on the other hand (**Figure 3** – strategy 2, TT vaccination). The CD27^hi^CD38^hi^ B cell subset (i) corresponds to a population of activated plasmablasts potentially enriched for antigen-specific Ig producing B cells [24], [25]. The CD27^+^CD38^lo/int^IgD^+^ (ii) and CD27^+^IgM^+^IgG^−^ (iv) B cell subsets contain the IgM-memory B cells [26], [27], whereas CD27^+^CD38^lo/int^IgD^−^ (iii) and CD27^+^IgM^−^IgG^+^ (v) B cell subsets contain the memory B cells that have undergone class-switch recombination. Still, the frequency of CD27^+^CD38^lo/int^IgD^−^ (iii) B cells was higher as compared to the frequency of CD27^+^IgM^−^IgG^+^ (v) B cells. This apparent discrepancy was explained by the fact that the large majority (>80%) of CD27^+^CD38^lo/int^IgD^−^ (iii) B cells were IgM^+^ B cells.

When comparing PBS-injected HIS mice to vaccinated non-responder and vaccinated responder animals, we did not observe any significant difference in the frequency of CD27^+^CD38^lo/int^ memory B cells (11.5±1.7% [n = 10], 8.2±0.7% [n = 20] and 13.0±2.1% [n = 14] of total B cells, respectively) and CD27^hi^CD38^hi^ plasmablasts (6.4±2.7%, 2.5±0.7% and 8.6±2.6% of total B cells, respectively), although the number of cells was increased for each of these subpopulations in the vaccinated animals, as expected from the enhanced number of total B cells (**Table 2**). We only observed a significant increase in the frequency of IgG^+^ B cells within the CD27^hi^CD38^hi^ plasmablast population of vaccinated responder HIS mice, as compared to PBS-injected animals (13.2±2.7% and 4.3±1.8% of CD27^hi^CD38^hi^ B cells, respectively; p = 0.0311).

In order to identify, isolate and immortalize the antigen-specific antibody-producing B cells, the aforementioned B cell subsets were transduced immediately after cell sorting with a retroviral vector encoding both human *BCL6* and *BCL-XL* [9], [19]. By ectopically expressing *BCL6* and *BCL-XL* in splenic or peripheral blood memory B cells and culturing them with factors produced by follicular helper T cells (CD40L and IL-21), we generated highly proliferative, BCR positive B cell lines that secrete Igs. Since these cells express BCL6, the differentiation of memory B cells to terminal plasma cells is blocked [28], [29], [30]. Therefore, the resulting B cells can expand extensively *in vitro* for long periods of time in presence of CD40L and IL-21, and provide a tool to generate antigen-specific human BCR-positive, antibody-secreting B cell lines.

The number of isolated cells from spleen and antigen-specific B cell clones that were generated with the *BCL6/BCL-XL* transduction approach is provided in the **Table S2**. Since the frequency of antigen-specific B cell clones was unknown, we started with micro-cell cultures ranging from 0.6 to 640 cells per well. The wells containing antigen-specific B cells – as determined by HBsAg-specific or TT-specific ELISA – were subsequently cultured by limiting dilution to obtain monoclonal B cell lines. Overall, we generated 15 anti-HBsAg IgM^+^ B cell clones from 3 HIS mice vaccinated with HBsAg, and 18 anti-TT IgM^+^ B cell clones from 3 HIS mice vaccinated with TT (**Table S2**). The estimated frequency of HBsAg-specific B cells (clones) in the HIS mice after vaccination was 1/350. The IgM secretion level of the B cell clones were in the range of 1 µg per 10^5^ cells over 3 days in culture, which was in a similar range of secretion (0.6–5 µg/10^5^ cells/3 days) to what was previously reported for B cell clones generated from human blood [9].

### Characterization of antibodies produced by the B cell clones

The IgM V_H_ regions of the BCR of the antigen-specific IgM^+^ B cell clones were sequenced. Overall, the BCR of HBsAg-specific and TT-specific B cell clones exhibited a V_H_ sequence close to the germ-line sequence, although limited frequencies of somatic hyper-mutations were observed (**Table S3** and **Table S4**). Somatic hyper-mutations were occasionally detected in all framework regions (FR) and complementary determining regions (CDR), and most of the BCR diversity was the result of N-additions in the CDR3 region. Based on the BCR sequence, we observed that 12 out the 15 anti-HBsAg IgM^+^ B cell clones were unique, as well as 5 out the 18 anti-TT IgM^+^ B cell clones (**Table S2, Table S3** and **Table S4**).

**Figure 4:**
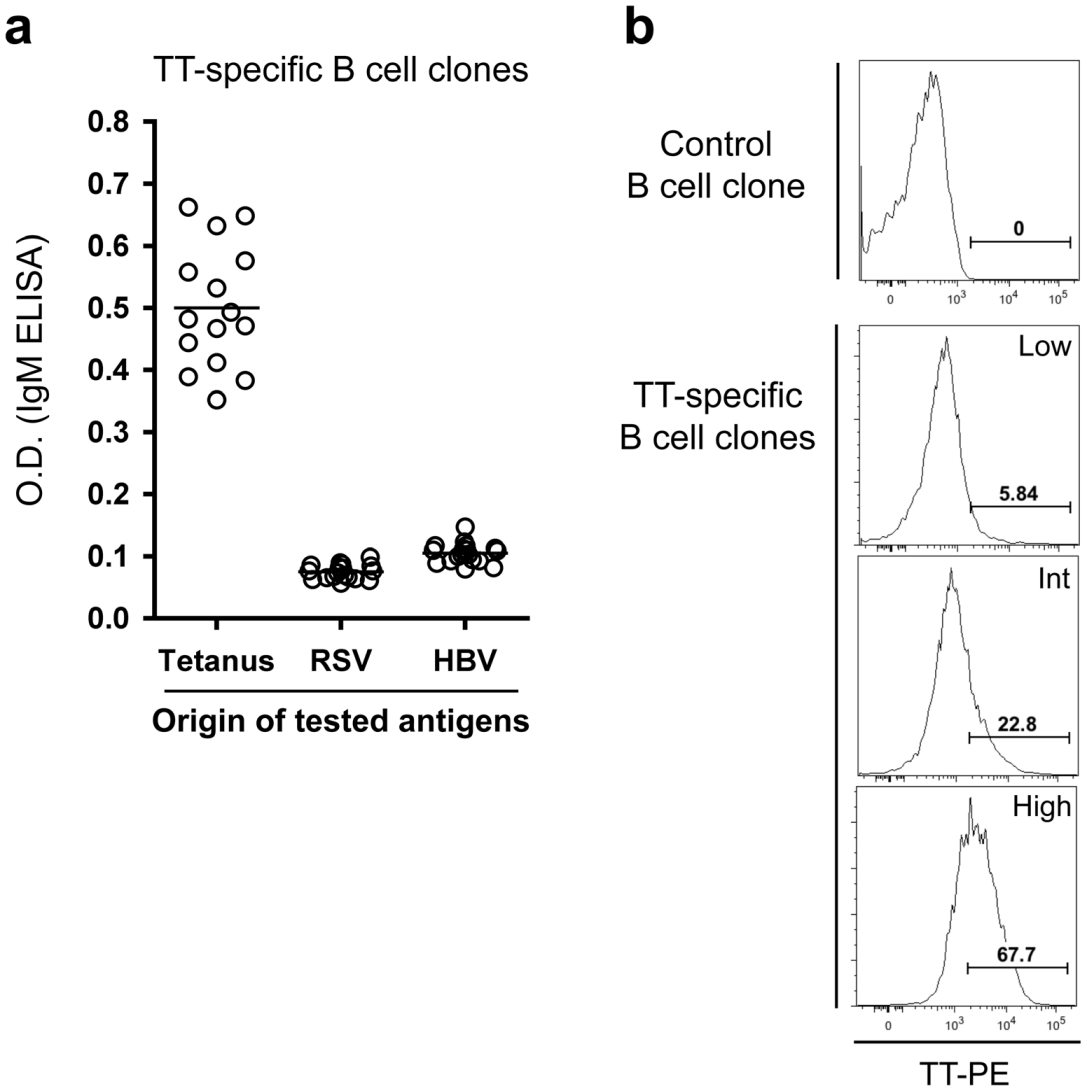
Characterization of the generated TT-specific human B cell clones. (A) After obtaining monoclonal TT-specific B cell lines, B cell clones were tested for cross-reactivity against unrelated antigens from HBV or RSV in an ELISA test. (B) The direct binding of phycoerythrin-labeled TT on TT ELISA positive B cell clones was determined by flow cytometry, and 3 sub-types of clones are observed: high, intermediate and low binding clones. Values in the histograms show the percentage of cells within the indicated gate.

The supernatants of TT-specific B cell clones were further tested for their capacity to recognize different antigens by ELISA. We observed that IgM mAbs did not cross-react with unrelated antigens (i.e., HBsAg and respiratory syncytial virus (RSV) antigens) (**Figure 4A**). The TT-specific B cell clones were also screened by flow cytometry for direct binding of the TT antigen labeled with a fluorochrome (**Figure 4B**). Interestingly, three types of clones that produced antibodies that gave a similar signal in ELISA were detected, with high, intermediate and low binding of the fluorescent TT antigen.

## Discussion

In the present work we established a new approach to generate fully human mAbs. We immortalized B cells from vaccinated HIS mice by transduction with *BCL6* and *BCL-XL* followed by expansion in presence of IL-21 and CD40L. Antigen-specific B cell clones were obtained that expressed the BCR on their cell surface and secreted antigen-specific antibodies. Similarly to methods based on the immortalization of human memory B cells from individuals that were either vaccinated or exposed to pathogens, our strategy exploits the antibody repertoire of human B cells which is likely to be different from that of B cells of mice expressing human Ig gene segments.

Naïve HIS mice display an extensive human IgM-expressing B cell repertoire. Based on the analysis of the length of the CDR3 regions, this IgM B cell repertoire is similar to the repertoire of healthy individuals. Thus, HIS mice have no obvious limitations for the generation of human IgM mAbs against any possible antigen. Upon intramuscular vaccinations with either TT or HBsAg, approximately 40% of the HIS mice were able to mount an antigen-specific antibody response. Human IgM-producing B cell lines against both antigens were obtained after isolation of memory B cells followed by *ex vivo* differentiation into plasmablast-like cells. It is important to highlight that the selection of the antigen-specific human B cell clones relied on relevant bioassays (e.g., ELISA or neutralization test). In contrast to EBV-based approaches, human B cell immortalization using transduction with *BCL-6* and *BCL-XL* preserves the expression of the BCR at the surface and antigen-specific B cell clones can also be selected by binding of labeled antigen to the BCR of immortalized memory B cells (e.g. by using a labeled antigen).

Even when IgG was used as a selection criterion, we were unable to establish antigen-specific IgG^+^ human B cell clones. The reason for this might be that T cell help in this system is suboptimal as indicated by the absence of antigen-specific T cell responses after vaccination (not shown). We also observed that the BCR of the B cell clones had a close to germ-line sequence, suggesting that also the induction of somatic hyper-mutation is sub-optimal in HIS mice. In our hands the great majority of the vaccinated HIS mice showed a defective formation of germinal centers [14], [16], which further explains the absence of antigen-specific Ig-class switched B cells. So far, humanized mouse models based on the transplantation of human HSC only – i.e. without additional human tissues – share these limitations, and immunization strategies result in the limited generation of class-switched antigen-specific B cell responses [14], [31], [32]. Similar patterns are observed in human HSC-transplanted immunodeficient mice infected with lymphotropic pathogens, such as HIV [33] or EBV [34], although Dengue virus infection in HIS mice was reported to induce an IgG response in a majority of the responder animals [35]. It is not clear why IgG antigen-specific responses are limited while serum IgG can accumulate efficiently, considering the low frequency of IgG^+^ B cells in HIS mice. It remains to be determined whether this apparent discrepancy might be explained by the conjunction of particularly effective IgG production on a cell basis by IgG^+^ B cells (which might occur in a T cell independent manner, such as in the case of the IgG3 subclass), long-term stability of human IgG in the HIS mouse serum as compared to human IgM, and/or defective survival of IgG^+^ B cells under specific conditions (e.g. after antigen-specific triggering of the BCR).

Although IgM mAbs might already be useful for some specific applications or could be modified by Ig class swapping to obtain IgG mAbs [36], optimized humanized mouse models with improved B cell function are highly desirable. One reason for the suboptimal interaction of T and B cells may be the poor survival resulting in a high turnover of human T cells (discussed in [10], [37]), making it very likely that procedures leading to improved accumulation of human T cells may promote B cell responses and isotype switching. It was already shown that human B cells undergoing isotype switching can be obtained in humanized mice, provided that a human environment supporting this process is present, e.g. in SCID mice transplanted with human fetal bones, thymus and lymph nodes [38]. Consistent with this notion, enhanced human peripheral T cell accumulation was observed in NOD/SCID mice transplanted with human bone marrow HSC, fetal liver and fetal thymus tissues (referred to as BLT mice), as compared to conventional humanized mouse systems [39]. Interestingly, BLT mice consistently generated an antigen-specific IgG response after HIV-infection [40]. Although it is yet unknown whether the isotype switch observed in BLT mice is truly T cell dependent, those data might support the idea that improved T cell homeostasis has a positive impact on B cell responses. To obtain humanized mouse models with improved B and T cell homeostasis, alternative strategies not relying on the transplantation of human fetal tissues – which are not necessarily easy to access to, for ethical, legal or practical reasons – will likely be favored in the future. The replacement of mouse genes involved in the hematopoietic system by their human equivalent is a valuable strategy to improve development, maintenance and/or function of several hematopoietic cell subsets in humanized mouse models, as shown with cytokines, such as IL-7 and Il-15 [15], [21], [41], [42], or MHC molecules (N.D.H and J.P.D., manuscript submitted) [43], [44]. The fact that the human CD47 was shown to be unable to properly interact with the mouse SIRPα indicates that re-introducing a functional phagocyte inhibition mechanism via the CD47/SIRPα signaling axis is another strategy of potential interest [45].

In conclusion, our results show using two standard vaccine antigens the general applicability of an innovative B cell immortalization method in combination with the HIS mouse model to generate human mAbs. Similarly to methods based on the immortalization of human memory B cells from vaccinated or convalescent individuals [9], our approach exploits the broad antibody repertoire of human B cells, overcoming the potential limitations of conventional humanized murine mAbs such as laboriousness or impaired biological properties, synthetic antibody libraries that require a known target antigen, and transgenic mice bearing the human Ig locus that have limited B cell repertoires. In addition, our method enables to exploit experimental infection models and immunization regimes that would be unethical or untenable in humans. Considering the upcoming advances in HIS mice models [37], this new approach will provide a powerful tool to generate human mAbs for either diagnostic or therapeutic purposes.

## Supporting Information

Figure S1 IgG/IgA B cell repertoire in naïve HIS mice. Similarly to Figure 1E, the naive IgG (A) and IgA (B) B cell repertoires of HIS (BALB-Rag/γ) mice were evaluated on splenocytes by performing a BCR immunoscope for each V_H_ family. The profiles obtained with control human PBMC are also shown.
(1.61 MB TIF)

Table S1 Human cell numbers measured in the BM of vaccinated HIS mice. Data from the TT and HBsAg vaccination experiments are pooled, and are presented as in the Table 2.
(0.06 MB DOC)

Table S2 Summary of B cell origin, sorted B cell numbers and antigen-specific B cell clone numbers. Memory B cell populations were sorted as shown in Figure 3. Limited dilutions of B cells transduced with *BCL-6* and *BCL-XL* were performed with 6.4 and 0.64 cells/well. After sub-cloning of the positive wells, we generated 15 IgM^+^ anti-HBsAg mAbs, of which 13 are unique (as determined by Ig-V_H_ sequence, see Table S3), and 18 IgM^+^ anti-TT mAbs, of which 5 are unique (see Table S4). In the case of HBsAg vaccination, the number of screened B cells was ((192*6.4)+(96*0.64))*3 = 3870, which eventually suggests that the frequency of HBsAg-specific B cells is at least 1/350 B cells.
(0.13 MB DOC)

Table S3 IgM V_H_ amino-acid sequence of generated HBsAg-specific B cell clones. The germ-line sequence is given for each V_H_ family, with indication of framework regions (FR) and complementary determining regions (CDR). Highlighted amino-acids correspond to N-additions (in the CDR3 region) and somatic hyper-mutation events, whether it results in a silent mutation (green) or not (red). Clones with identical BCR sequences are grouped together.
(0.02 MB PDF)

Table S4 IgM V_H_ amino-acid sequence of generated TT-specific B cell clones. Data are presented as in the Table S2.
(0.01 MB PDF)
