# Supplementary figures and images for: Generation of Human Antigen-Specific Monoclonal IgM Antibodies Using Vaccinated “Human Immune System” Mice
Source: PLoS One. 2010 Oct 4;5(10):e13137. doi: 10.1371/journal.pone.0013137 (PMC2949385; doi:10.1371/journal.pone.0013137)

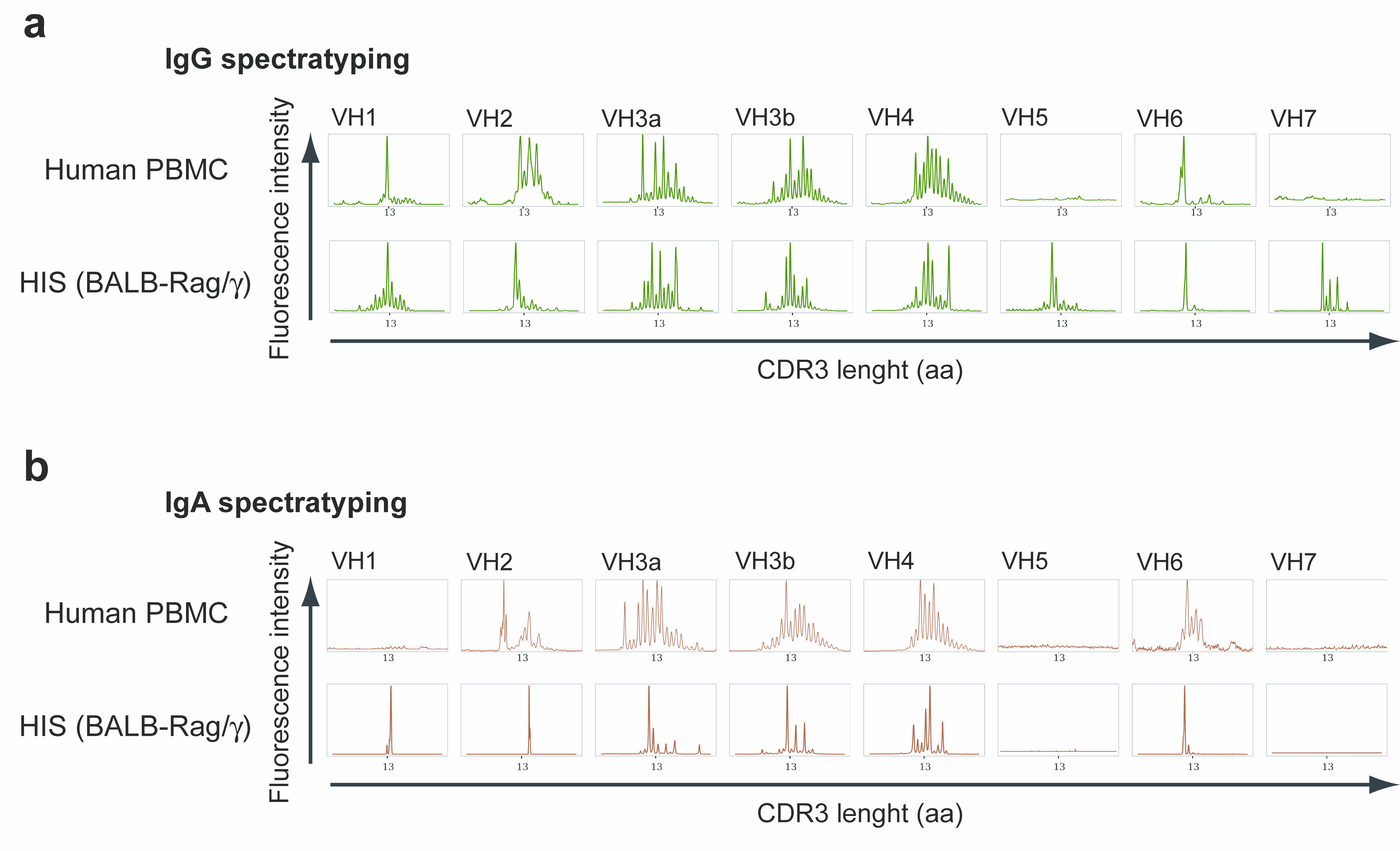

Supplement: Figure S1 — IgG/IgA B cell repertoire in naïve HIS mice. Similarly to Figure 1E, the naive IgG (A) and IgA (B) B cell repertoires of HIS (BALB-Rag/γ) mice were evaluated on splenocytes by performing a BCR immunoscope for each VH family. The profiles obtained with control human PBMC are also shown. (1.61 MB TIF) [file pone.0013137.s001.tif]
