# Supplementary material for: Generation of Human Antigen-Specific Monoclonal IgM Antibodies Using Vaccinated “Human Immune System” Mice
Source: PLoS One. 2010 Oct 4;5(10):e13137. doi: 10.1371/journal.pone.0013137 (PMC2949385; doi:10.1371/journal.pone.0013137)
Supplement: Table S1 — Human cell numbers measured in the BM of vaccinated HIS mice. Data from the TT and HBsAg vaccination experiments are pooled, and are presented as in the Table 2. (0.06 MB DOC) [file pone.0013137.s002.doc]

**Table S1. Human cell numbers measured in the BM of vaccinated HIS mice.**

|  | **Human cells (CD45^+^)** | | |
| --- | --- | --- | --- |
| **Groups** | **Total** | **B cells (CD19^+^)** | **T cells (CD3^+^)** |
|  | **Absolute number (x10^5^)** | **Absolute number (x10^5^)** | **Absolute number (x10^5^)** |
| **Controls (n=10)** | 3.67 ± 1.54 | 0.39 ± 0.20 | 2.85 ± 1.38 |
| **Vaccinated (n=34)** | 34.40 ±7.98* | 27.11 ± 7.30* | 2.81 ± 0.66  *n.s.* |
| *responders (n=14)* | 38.16 ±13.29* | 29.08 ± 12.19* | 4.12 ± 1.30  *n.s.* |
| *non-responders (n=20)* | 31.78 ±10.13* | 25.73 ± 9.27** | 1.89 ± 0.60  *n.s.* |

Data from the TT and HBsAg vaccination experiments are pooled, and are presented as in the **Table 2**.
