# Supplementary material for: Generation of Human Antigen-Specific Monoclonal IgM Antibodies Using Vaccinated “Human Immune System” Mice
Source: PLoS One. 2010 Oct 4;5(10):e13137. doi: 10.1371/journal.pone.0013137 (PMC2949385; doi:10.1371/journal.pone.0013137)
Supplement: Table S2 — Summary of B cell origin, sorted B cell numbers and antigen-specific B cell clone numbers. Memory B cell populations were sorted as shown in Figure 3. Limited dilutions of B cells transduced with BCL-6 and BCL-XL were performed with 6.4 and 0.64 cells/well. After sub-cloning of the positive wells, we generated 15 IgM+ anti-HBsAg mAbs, of which 13 are unique (as determined by Ig-VH sequence, see Table S3), and 18 IgM+ anti-TT mAbs, of which 5 are unique (see Table S4). In the case of HBsAg vaccination, the number of screened B cells was ((192*6.4)+(96*0.64))*3 = 3870, which eventually suggests that the frequency of HBsAg-specific B cells is at least 1/350 B cells. (0.13 MB DOC) [file pone.0013137.s003.doc]

**Table S2. Summary of B cell origin, sorted B cell numbers and antigen-specific B cell clone numbers.**

| **HIS mouse number** | **Organ** | **Sorted B cell population** | **# sorted cells** | **# retrieved clones** | **# unique clones** | **Clones ID** |
| --- | --- | --- | --- | --- | --- | --- |
|  |  |  |  |  |  |  |
| **HBV-2 (#6)** | SPL | CD27^+^IgD^+^ | 28.400 | 1 | 1 | Γ1-4 |
| **HBV-3 (#14)** | SPL | CD27^+^IgD^+^ | 3.645 | 3 | 2 | γ2-15; 2β2-H8-2; 2β2-D6-11 |
| **HBV-3 (#14)** | SPL | CD27^+^IgD^-^ | 10.700 | 2 | 2 | 3β2-F2-2; 3β2-G1-7 |
| **HBV-4 (#23)** | SPL | CD27^+^IgD^+^ | 11.600 | 7 | 6 | γ6-7; γ6-14; γ6-21; 6β2-D4-3; 6β1-E3-10; 5β1-E3-5; 6β2-G11-6 |
| **HBV-4 (#23)** | SPL | CD27^hi^CD38^hi^ | 20.700 | 2 | 2 | γ5-8; 5β2-D5-5 |
|  |  |  |  |  |  |  |
| **TT-1 (#7)** | SPL | CD27^+^IgM^+^IgG^-^ | 29.500 | 1 | 1 | 3F7 |
| **TT-2 (#10)** | SPL | CD27^+^IgM^+^IgG^-^ | 18.030 | 4 | 2 | 6E7; 8B11; 8D11; 10C6 |
| **TT-4 (#17)** | SPL | CD27^+^IgM^+^IgG^-^ | 5.360 | 13 | 2 | 11D10; 11G9; 12B5; 12C6; 12G5; 12G11; 13C5; 13E6; 13F5; 14H7; 14H11; 15C9; 15D11 |

Memory B cell populations were sorted as shown in Figure 3. Limited dilutions of B cells transduced with BCL-6 and BCL-XL were performed with 6.4 and 0.64 cells/well. After sub-cloning of the positive wells, we generated 15 IgM^+^ anti-HBsAg mAbs, of which 13 are unique (as determined by Ig-V_H_ sequence, see **Table S3**), and 18 IgM^+^ anti-TT mAbs, of which 5 are unique (see **Table S4**). In the case of HBsAg vaccination, the number of screened B cells was ((192*6.4)+(96*0.64))*3=3870, which eventually suggests that the frequency of HBsAg-specific B cells is at least 1/350 B cells.
