# Supplementary material for: Generation of Human Antigen-Specific Monoclonal IgM Antibodies Using Vaccinated “Human Immune System” Mice
Source: PLoS One. 2010 Oct 4;5(10):e13137. doi: 10.1371/journal.pone.0013137 (PMC2949385; doi:10.1371/journal.pone.0013137)
Supplement: Table S3 — IgM VH amino-acid sequence of generated HBsAg-specific B cell clones. The germ-line sequence is given for each VH family, with indication of framework regions (FR) and complementary determining regions (CDR). Highlighted amino-acids correspond to N-additions (in the CDR3 region) and somatic hyper-mutation events, whether it results in a silent mutation (green) or not (red). Clones with identical BCR sequences are grouped together. (0.02 MB PDF) [file pone.0013137.s004.pdf]

Table S3. IgM V_H amino-acid sequence of generated HBsAg-specific B cell clones.

CLONES	-----------FR1-----------	---CDR1---	-------FR2-------	---CDR2---	------------------FR3------------------	------CDR3-------	----FR4----
**VH3-73**	**EVQLVESGGGLVQPGGSLKLSCAAS**	**GFTFSGSA**	**MHWVRQASGKGLEWVGR**	**IRSKANSYAT**	**AYAASVK GRFTISRDDSKNTAYLQMNSLKTEDTAVYYC**	**TR**	**WGQGTLVTVSS**
**6β2-D4-3**	GGGLVQPGGSLKLSCAAS	GFTFSGSA	MHWVRQASGKGLEWVGR	IRSKANSYAT	AYAASVK GRFTISRDDSKNTAYLQMNSLKTEDTAVYYC	TSRKSSSSDY	WGQGTLVTVSS
**6β1-E3-10**	EVQLVESGGGLVQPGGSLKLSCAAS	GFTFSGSA	MHWVRQASGKGLEWIGR	IGSKANSYAT	AYAAPVK GRFTISRDDSKNTAYLQMNSLKTEDTAVYYC	TSRKSSSSDY	WGQGTLVTVSS
**3β2-F2-2**	EVQLVESGGGLVQPGGSLKLSCAAS	GFTFSGSA	MHWVRQASGKGLEWVGR	IRSKPNSYAT	AYAASVK GRFTISRDDSKNTAYLQMNSLKTEDTAVYYC	TRLSGRGVDY	WGQGTLVTVSS
**6β2-G11-6**	EVQLVESGGGLVQPGGSLKLSCAAS	GFTFSGSA	MHWVRQASGKGLEWVGR	IRSKANSYAT	AYAASVK GRFTISRDDSKNTAYLQMNSLKTEDTAVYYC	TRKSSSSDY	WGQGTLVTVSS
**5β2-D5-5**	EVQLVESGGGLVQPGGSLKLSCAAS	GFTFSGSA	MHWVRQASGKGLEWVGR	IRSKANSYAT	AYAASVK GRFTISRDDSKNTAYLQMNSLKTEDTAVYYC	TRTYSSSWYFDY	WGQGTLVTVSS
**γ5-8**	EVQLVESGGGLVQPGGSLKLSCAAS	GFTFSGSA	MHWVRQASGKGLEWVGR	IRSKANSYAT	AYAASVK GRFTISRDDSKNTAYLQMNSLKTEDTAVYYC	TRTYSSSWYFDY	WGQGTLVTVSS
**γ6-7**	EVQLVESGGGLVQPGGSLKLSCAAS	GFTFSGSA	MHWVRQASGKGLEWVGR	IRSKANSYAT	AYAASVK GRFTISRDDSKNTAYLQMNSLKTEDTAVYYC	TRRGYYGSGSYYGDY	WGQGTLVTVSS
**5β1-E3-5**	EVQLVESGGGLVQPGGSLKLSCAAS	GFTFSGSA	MHWVRQASGKGLEWVGR	IRSKANSYAT	AYAASVK GRFTISRDDSKNTAYLQMNSLKTEDTAVYYC	TRRGYYGSGSYYGDY	WGQGTLVTVSS
**VH4-34**	**QVQLQQWGAGLLKPSETLSLTCAVY**	**GGSFSGYY**	**WSWIRQPPGKGLEWIGE**	**INHSGST**	**NYNPSLK SRVTISVDTSKNQFSLKLSSVTAADTAVYYC**	**AR**	**WGQGTLVTVSS**
**2β2-D6-11**	QVQLQQWGAGLLKPSETLSLTCAVY	GGSFSVYY	WSWIRQPPGKGLEWIGK	INHSGST	NYNPSLK SRVTISVDTSKNQFSLKLSSVTAADTAVYYC	ARGFHY	WGQGTLVTVSS
**VH3-30**	**QVQLVESGGGVVQPGRSLRLSCAAS**	**GFTFSSYG**	**MHWVRQAPGKGLEWVAV**	**ISYDGSNK**	**YYADSVK GRFTISRDNSKNTLYLQMNSLRAEDTAVYYC**	**AR**	**WGQGTLVTVSS**
**3β2-G1-7**	QVQLVESGGGVVQPGRSLRLSCAAS	GFTFSSYG	MHWVRQAPGKGLEWVAV	ISYDGSNK	YYADSVK GRFTISRDNSKNTLYLQMNSLRAEDTAVYYC	AKAVVDRARDGYNLGY	WGQGTLVTVSS
**γ6-14**	PGRSLRLSCAAS	GFTFSTYA	MHWVRQAPGKGLEWVAV	ISYDGSNK	YYADSVK GRFTISRDNSKNTLYLQMNSLRAEDTAVYYC	ARGTYYGSGIGFDY	WGQGTLVTVSS
**VH3-15**	**EVQLVESGGGLVKPGGSLRLSCAAS**	**GFTFSNAW**	**MSWVRQAPGKGLEWVGR**	**IKSKTDGGTT**	**DYAAPVK GRFTISRDDSKNTLYLQMNSLKTEDTAVYYC**	**TT**	**WGQGTLVTVSS**
**2β2-H8-2**	EVQLVESGGGLVKPGGSLRLSCAAS	GFTFSNAW	MSWVRQAPGKGLEWVGR	IKSKTDGGTT	DYAAPVK GRFTISRDDSKNTLYLQMNSLKTEDTAVYYC	LINWGIRD	WGQGTLVTVSS
**γ2-15**	GSLRLSCAAS	GFTFSNAW	MSWVRQAPGKGLEWVGR	IKSKTDGGTT	DYAAPVK GRFTISRDDSKNTLYLQMNSLKTEDTAVYYC	LINWGIRD	WGQGTLVTVSS
**VH5-51**	**EVQLVQSGAEVKKPGESLKISCKGS**	**GYSFTSYW**	**IGWVRQMPGKGLEWMGI**	**IYPGDSDT**	**RYSPSFQ GQVTISADKSISTAYLQWSSLKASDTAMYYC**	**AR**	**WGQGTLVTVSS**
**γ1-4**	EVQLVQSGAEVKKPGESLKISCKGS	GYSFTTYW	IGWVRQMPGKGLEWMGI	IYPGDSDT	RYSPSFQ GQVTISADKSISTAYLQWSSLKASDTAMYYC	ARHSEYYYDSSGYYLDY	WGQGTLVTVSS
**VH5-A**	**EVQLVQSGAEVKKPGESLRISCKGS**	**GYSFTSYW**	**ISWVRQMPGKGLEWMGR**	**IDPSDSYT**	**NYSPSFQ GHVTISADKSISTAYLQWSSLKASDTAMYYC**	**AR**	**WGQGTLVTVSS**
**γ6-21**	EVQLVQSGAEVKKPGESLRISCKSS	GYSFTSYW	ISWVRQMPGKGLEWMGR	IDPSDSYT	KYSPSFQ GHVIISADKSISTAYLQWSSLKASDTAMYYC	ARHLREAVADFPMDV	WG

The germ-line sequence is given for each V_H family, with indication of framework regions (FR) and complementary determining regions (CDR). Highlighted amino-acids correspond to N-additions (in the CDR3 region) and somatic hyper-mutation events, whether it results in a silent mutation (green) or not (red). Clones with identical BCR sequences are grouped together.
