# Supplementary material for: Generation of Human Antigen-Specific Monoclonal IgM Antibodies Using Vaccinated “Human Immune System” Mice
Source: PLoS One. 2010 Oct 4;5(10):e13137. doi: 10.1371/journal.pone.0013137 (PMC2949385; doi:10.1371/journal.pone.0013137)
Supplement: Table S4 — IgM VH amino-acid sequence of generated TT-specific B cell clones. Data are presented as in the Table S2. (0.01 MB PDF) [file pone.0013137.s005.pdf]

Table S4. IgM V_H amino-acid sequence of generated TT-specific B cell clones.

CLONES	-----------FR1-----------	---CDR1---	-------FR2-------	---CDR2---	-----------------FR3-----------------	--------CDR3--------	----FR4----
**VH3-30**	**QVQLVESGGGVVQPGRSLRLSCAAS**	**GFTFSSYG**	**MHWVRQAPGKGLEWVAV**	**ISYDGSNK**	**YYADSVKGRFTISRDNSKNTLYLQMNSLRAEDTAVYYC**	**AR**	**GQGTTVTVS**
**12G11**	QVQLVESGGGVVQPGRSLRLSCAAS	GFTFSSY**A**	MHWVRQAPGKGLEWVAV	ISYDGSNK	YYADSVKGRFTISRDNSKNTLYLQMNSLRAEDTAVYYC	AR**TYGGKASIAARRDHYGMDVW**	GQGTTVTVS
**12C6**	GGGVVQPGRSLRLSCAAS	GFTFSSY**A**	MHWVRQAPGKGLEWVAV	ISYDGSNK	YYADSVKGRFTISRDNSKNTLYLQMNSLRAEDTAVYYC	AR**TYGGKASIAARRDHYGMDVW**	GQGTTVTVS
**15D11**	QVQLVESGGGVVQPGRSLRLSCAAS	GFTFSSY**A**	MHWVRQAPGKGLEWVAV	ISYDGSNK	YYADSVKGRFTISRDNSKNTLYLQMNSLRAEDTAVYYC	AR**TYGGKASIAARRDHYGMDVW**	GQGTTVTVS
**14H7**	QVQLVESGGGVVQPGRSLRLSCAAS	GFTFSSY**A**	MHWVRQAPGKGLEWVAV	ISYDGSNK	YYADSVKGRFTISRDNSKNTLYLQMNSLRAEDTAVYYC	AR**TYGGKASIAARRDHYGMDVW**	GQGTTVTVS
**13C5**	QVQLVESGGGVVQPGRSLRLSCAAS	GFTFSSY**A**	MHWVRQAPGKGLEWVAV	ISYDGSNK	YYADSVKGRFTISRDNSKNTLYLQMNSLRAEDTAVYYC	AR**TYGGKASIAARRDHYGMDVW**	GQGTTVTVS
**15C9**	QVQLVESGGGVVQPGRSLRLSCAAS	GFTFSSY**A**	MHWVRQAPGKGLEWVAV	ISYDGSNK	YYADSVKGRFTISRDNSKNTLYLQMNSLRAEDTAVYYC	AR**TYGGKASIAARRDHYGMDVW**	GQGTTVTVS
**13E6**	QVQLVESGGGVVQPGRSLRLSCAAS	GFTFSSY**A**	MHWVRQAPGKGLEWVAV	ISYDGSNK	YYADSVKGRFTISRDNSKNTLYLQMNSLRAEDTAVYYC	AR**TYGGKASIAARRDHYGMDVW**	GQGTTVTVS
**11D10**	QVQLVESGGGVVQPGRSLRLSCAAS	GFTFSSY**A**	MHWVRQAPGKGLEWVAV	ISYDGSNK	YYADSVKGRFTISRDNSKNTLYLQMNSLRAEDTAVYYC	AR**TYGGKASIAARRDHYGMDVW**	GQGTTVTVS
**11G9**	QVQLVESGGGVVQPGRSLRLSCAAS	GFTFSSY**A**	MHWVRQAPGKGLEWVAV	ISYDGSNK	YYADSVKGRFTISRDNSKNTLYLQMNSLRAEDTAVYYC	AR**TYGGKASIAARRDHYGMDVW**	GQGTTVTVS
**14H11**	PGRSLRLSCAAS	GFTFSSY**A**	MHWVRQAPGKGLEWVAV	ISYDGSNK	YYADSVKGRFTISRDNSKNTLYLQMNSLRAEDTAVYYC	AR**TYGGKASIAARRDHYGMDVW**	GQGTTVTVS
**12G5**	QVQLVESGGGVVQPGRSLRLSCAAS	GFTFSSY**A**	MHWVRQAPGKGLEWVAV	ISYDGSNK	YYADSVKGRFTISRDNSKNTLYLQMNSLRAEDTAVYYC	AR**TYGGKASIAARRDHYGMDVW**	GQGTTVTVS
**12B5**	QVQLVESGGGVVQPGRSLRLSCAAS	GFTFSSY**A**	MHWVRQAPGKGLEWVAV	ISYDGSNK	YYADSVKGRFTISRDNSKNTLYLQMNSLRAEDTAVYYC	AR**TYGGKASIAARRDHYGMDVW**	GQGTTVTVS
**10C6**	QVQLVESGGGVVQPGRSLRLSCAAS	GFTFSSYG	MHWVRQAPGKGLEWVAV	ISYDGSNK	YYADSVKGRFTISRDNSKNTLYLQMNSLRAEDTAVYYC	A**KGGWNYYGSGSYYW**	GQGTTVTVS
**8B11**	QVQLVESGGGVVQPGRSLRLSCAAS	GFTFSSYG	MHWVRQAPGKGLEWVAV	ISYDGSNK	YYADSVKGRFTISRDNSKNTLYLQMNSLRAEDTAVYYC	A**KGGWNYYGSGSYYW**	GQGTTVTVS
**8D11**	QVQLVESGGGVVQPGRSLRLSCAAS	GFTFSSYG	MHWVRQAPGKGLEWVAV	ISYDGSNK	YYADSVKGRFTISRDNSKNTLYLQMNSLRAEDTAVYYC	A**KGGWNYYGSGSYYW**	GQGTTVTVS
**6E7**	QVQLVESGGGVVQPGRSLRLSCAAS	GFTFSSY**A**	MHWVRQAPGKGLEWVAV	ISYDGSNK	YYADSVKGRFTISRDNSKNTLYLQM**T**SLRAEDTAVYYC	AR**APGGRGVIIWAFDYW**	GQGTTVTVS
**3F7**	GVVQPGRSLRLSCAAS	GFTFSSYG	MHWVRQAPGKGLEWVAV	ISYDGSNK	YYADSVKGRFTISRDNSKNTLYLQMNSLRAEDTAVYYC	A**KDRTAAAGTPKRSWFDPW**	GQGTTLVTVS
**13F5**	QVQLVESGGGVVQPGRSLRLSCAAS	GFTFSSYG	MHWVRQAPGKGLEWVAV	ISYDGSNK	YYADSVKGRFTISRDNSKNTLYLQMNSLRAEDTAVYYC	A**KQAGIAVAGSFDYW**	GQGTTLVTVS

Data are presented as in the **Table S2**.
